# MpUL-multi: Software for Calculation of Amyloid Fibril Mass per Unit Length from TB-TEM Images

**DOI:** 10.1038/srep21078

**Published:** 2016-02-12

**Authors:** Matthew G. Iadanza, Matthew P. Jackson, Sheena E. Radford, Neil A. Ranson

**Affiliations:** 1 Astbury Centre for Structural Molecular Biology, School of Molecular and Cellular Biology, University of Leeds, Leeds, LS2 9JT. UK

## Abstract

Structure determination for amyloid fibrils presents many challenges due to the high variability exhibited by fibrils and heterogeneous morphologies present, even in single samples. Mass per unit length (MPL) estimates can be used to differentiate amyloid fibril morphologies and provide orthogonal evidence for helical symmetry parameters determined by other methods. In addition, MPL data can provide insight on the arrangement of subunits in a fibril, especially for more complex fibrils assembled with multiple parallel copies of the asymmetric unit or multiple twisted protofilaments. By detecting only scattered electrons, which serve as a relative measure of total scattering, and therefore protein mass, dark field imaging gives an approximation of the total mass of protein present in any given length of fibril. When compared with a standard of known MPL, such as Tobacco Mosaic Virus (TMV), MPL of the fibrils in question can be determined. The program suite MpUL-multi was written for rapid semi-automated processing of TB-TEM dark field data acquired using this method. A graphical user interface allows for simple designation of fibrils and standards. A second program averages intensities from multiple TMV molecules for accurate standard determination, makes multiple measurements along a given fibril, and calculates the MPL.

Amyloid fibrils are the result of the self assembly of folded, partially folded, or denatured proteins into large fibular structures in a sequence dependent manner^1^. *In vivo* formation of amyloid fibrils under physiological conditions is associated with well known disease states such as Alzheimer’s disease^2^, Parkinson’s disease^3^,^4^, and Type II Diabetes Mellitus^5^, as well as numerous less common disease states^6^. Fibrils formed from both natural and designed proteins and peptides have also shown promise as platforms for nanoengineered devices^7^.

Determination of amyloid fibril structures by electron microscopy is generally performed using helical reconstruction methods such as iterative real space helical reconstruction (IHRSR)^8^ or using programs such as SPRING^9^, or FREALIX^10^. All such methods require accurate knowledge of the helical symmetry of the fibril in question, and this can be difficult to determine for biological assemblies, owing to the lack of distinct structural features in noisy EM images. This problem is exacerbated when studying amyloid fibrils owing to their typically large helical repeat and structural heterogeneity^11^. Studies aiming to determine amyloid fibril structure therefore typically deduce this information by combining methods such as examination of the Bessel orders present in Fourier transforms of cryo-EM averages or negative stain images^12^, or direct measurements from cryo-EM and/or negative stain EM^13^. Estimates of mass per unit length (MPL) can provide invaluable orthogonal evidence about the helical symmetry of an object and give an independent validation of parameters determined by other methods. They can also provide insight into the arrangement of subunits in the fibril, helping build pseudo-atomic models for fibril architectures using relatively low-resolution EM density^14^. This may be especially interesting for more complex fibrils assembled with multiple copies of the asymmetric unit or multiple twisted protofilaments.

Traditionally, MPL has been determined using scanning transmission electron microscopy (STEM). STEM allows for highly accurate mass determination, without internal standards, using a calibrated detector^15^. This eliminates inaccuracy due to both variation in the standards and error in their measurements. Advanced software exists for processing STEM data, which further improves accuracy by compensating for beam induced mass loss and dynamic scattering^16^. MPL measurements by STEM have been used to differentiate fibril morphologies and validate biochemical models for amyloid fibrils composed of a variety of proteins including Calcitonin^17^, Amyloid beta^18^, and IAPP^19^,^20^.

Whilst STEM using a calibrated detector remains the “gold standard” for determination of MPL, Chen *et al.*^21^ presented an alternative method using tilted beam transmission electron microscopy (TB-TEM), which can be performed with a standard transmission EM. This ability to use standard TEM makes MPL estimation far more accessible. By using dark field imaging, TB-TEM detects only scattered electrons, the number of which is proportional to total scattering and therefore to protein mass. When compared with a standard of known MPL, the MPL of the fibrils in question can be determined. Although not as accurate as MPL determination by STEM^15^ this allows for MPL determination with sufficient accuracy to give a reasonable estimation of the number of monomers present in a given length of fibril and can be used to distinguish some varying fibril morphologies^21^. Here we present a new software tool, MpUL-multi, which allows the rapid semi-automated processing of TB-TEM data with little or no expert knowledge, to allow rapid estimation of MPL for fibrilar biological assemblies.

## Program

MpUL-multi uses an extension of the method described by Chen *et al.* to determine sample and background intensity for a standard protein of known MPL and the fibril of interest. The inputs for MpUL-multi are TB-TEM dark field images that contain the fibrils of interest and a standard of known MPL, such as tobacco mosaic virus (TMV). After performing background subtraction, the intensity of the fibrils is compared with the intensity of the standard, allowing for estimation of the MPL of the unknown fibril. The program allows for the averaging of values from multiple standards to reduce variability due to differences in the intensity measured from the individual TMV molecules.

**Figure 1:**
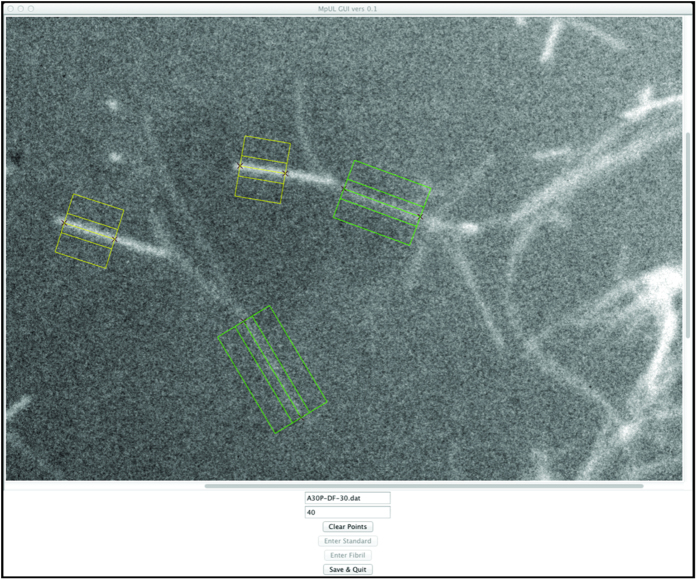
MpUL-Multi GUI. An example of the GUI window, zoomed in to show selected standards (yellow) and fibrils (green). The selection boxes delineate the areas used to determine fibril intensity and background intensity.

TMV standards and multiple areas along a fibril are defined by specifying the start and end coordinates for lines along the fibril long axis, and defining its width using the GUI (Fig. 1). Pixel values along one-pixel wide lines perpendicular to the fibril axis are then measured, reporting a fibril intensity and two background intensity measurements. These values are then used with the measurements from the TMV standards in the same image to determine the MPL for the fibril as:


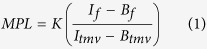


Where I_f_ and I_tmv_ are the uncorrected intensity measurements for the fibril and TMV standard, B_f_ and B_tmv_ are corresponding background intensity measurements for each intensity measurement, and K is the MPL of TMV, taken form the literature as 131 kDa nm^−1^ 22. The individual 1 px wide measurements from all subregions of the fibrils are then combined to calculate the mean MPL.

**Figure 2:**
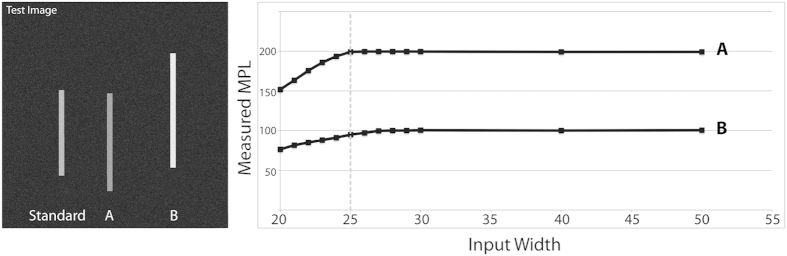
Testing robustness of fibril width settings. A test image was created containing three bars on a black background, each with a width of 25 pixels. The bars have three different pixel intensity values: 131 to represent the TMV standard, 100 for bar A, and 200 for bar B. The entire image has gaussian noise with a standard deviation of 25 applied. Measurements were made along bars A and B in the test image using MpUL-Multi with the width measurement setting varying from 20 to 40 px and the resulting MPL measurements reported. The actual width of the test bars (25 px) is designated by a dotted line. The systematic underestimation of MPL when the specified width is too small is apparent. The measurements become more robust as the width parameter is increased beyond the actual width of the fibril. A slight underestimation of MPL at the actual width due to imperfect centering on bar B is remedied as the width grows larger than the actual fibril width.

The program does not require an exact determination of the width of the fibrils being measured, but instead requires only that the defined width be wider than the actual fibril width and is robust in regard to small errors in centering the line along which the measurements are made on the fibrils. This was tested by making a series of measurements of various widths on a test image containing bars with standardized widths and pixel values (Fig. 2).

## Validation and Testing

MPL measurements were performed on two fibular structures of known MPL; F-actin and β_2_-microglobin (β_2_m) formed *in vitro* at pH 2.0^13^, as well as three fibrils of unknown MPL; full length wild-type α-synuclein (α-syn), and two disease related α-synuclein mutants: A30P and A53T^23^.

## Materials and Methods

### Preparation of amyloid fibrils

Full-length recombinant β_2_m was expressed in *E. coli* and purified as described previously^24^. The protein was dissolved in buffer containing 10 mM sodium phosphate buffer (pH 2.0) and 50 mM NaCl, filtered through a 0.22 μm Millipore syringe filter, and adjusted to 120 mM final concentration. Fibrils were formed by seeding with 0.1% w/w of short preformed fibrils fragmented by mechanical agitation as in^25^. The mixture was incubated quiescently at 25°C for 48 h to allow the growth of long fibrils. Wild-type α-synuclein (WT α-syn), α-synuclein A30P (α-syn A30P), and α-synuclein A53T (α-syn A53T) were expressed recombinantly in *E. coli* and purified by ammonium sulfate precipitation followed by size exclusion chromatography. Monomeric protein was dissolved in 20 mM TRIS HCl (pH 7.4) with 100 mM NaCl, filtered through a 0.22 μm Millipore syringe filter and diluted to a final concentration of 1 mg/ml. Fibrils were allowed to form *de novo* by nucleation with shaking for 7 days at room temperature^26^. F-actin was prepared by diluting a 4.0 mg/ml solution of G-actin in 2 mM TRIS-HCl at pH 8.0 with 0.2 mM CaCl_2_, 0.5 mM DTT, and 0.2 mM ATP into buffer containing 10 mM MOPS (pH 7.0), 50 mM KCl, 1 mM MgCl_2_, and 1 mM EGTA as in^27^.

### Electron microscopy grid preparation

Copper 200-mesh EM grids were floated on a layer of colloidion (Sigma, USA) on Milli-Q water. The colloidion coated grids were removed, dried, and coated with a 10 nm thick layer of carbon, and glow discharged immediately before use. 4 μl of fibril solution was then applied to the grid for 30 seconds. The grid was then blotted with filter paper and washed with Milli-Q water. 4 μl of 0.1 mg/ml TMV in 25 mM TRIS HCl (pH 7.5) was then applied. The grid was then blotted with filter paper, washed twice with Milli-Q water, and air-dried.

### Electron microscopy

**Figure 3:**
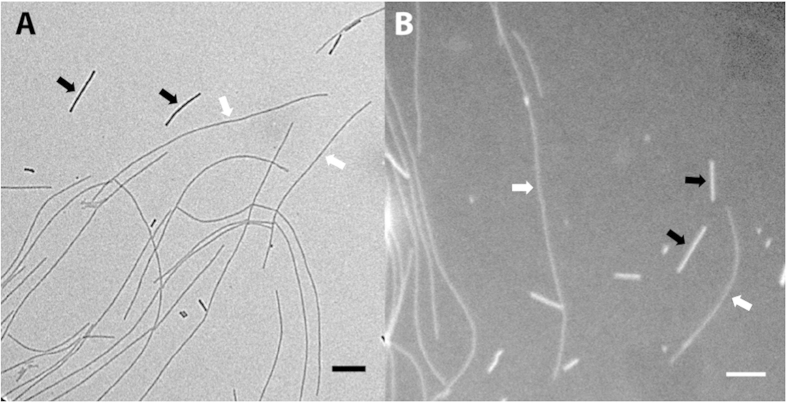
Bright field and dark field imaging of unstained fibrils. Images of wild type α-synuclein and TMV standards made with (**A**) bright field and (**B**) dark field illumination. Examples of TMV standards and amyloid fibrils are indicated by black and white arrows respectively. Scale bars are 200 nm.

Dark field imaging of the unstained grids was performed as in^21^ using a FEI T12 Technai TEM operated at 80 kV. Regions containing both TMV and fibrils were identified and the microscope focused using bright field illumination (Fig. 3A). The electron beam was then tilted to −1° and beam intensity adjusted to give even illumination and maximum intensity over the entire image. Images were recorded on an UltraScan US1000XP CCD (Gatan Inc, USA.) using a 10 second exposure (Fig. 3B).

### Image processing

The images were converted to 8 bit TIF format with ImageJ and processed using MpUL-multi. All available molecules of TMV in each image were selected to serve as the MPL standard. Individual WT α-syn, α-syn A30P, α-syn A53T, F-actin, and β_2_m fibrils were measured in separate micrographs. In all cases fibrils that appeared to be of the lowest order were selected for measurements. Higher-order structures consisting of multiple twisted fibrils were excluded.

### Program availability

MpUL-multi and the MpUL-GUI are distributed under the GNU General Public License^33^ and available (with test data and comprehensive instructions) from the Collaborative Computational Project for Electron cryo-Microscopy (CCP-EM; http://www.ccpem.ac.uk/download.php) The programs are written in python 2.7.1 and require the “math”, “TkInter”, “os”, “numpy”, and “sys” standard modules. A local copy of ImageJ (or FIJI) is also required.

## Results and Discussion

**Figure 4:**
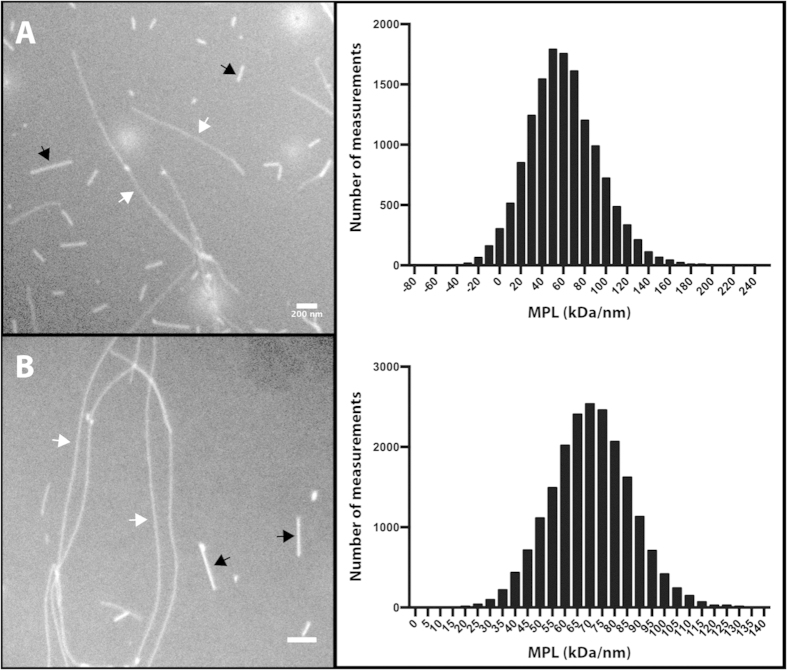
Example micrographs and measurements. Example dark field images and histograms showing all measurements for (**A**) β2-microgloblin and (**B**) Wild type α-synuclein fibrils. Examples of TMV standards and amyloid fibrils are indicated by black and white arrows respectively. Scale bars are 200 nm.

To benchmark the accuracy of the MpUL-multi programs we determined the mean MPL for two samples for which MPL estimates already exist. (Table 1) The MPL of F-actin was calculated as 17 ± 5 kDa.nm^−1^ (n = 7 fibrils, 814 measurements) which corresponds well to the theoretical value of 15.6 kDa nm^−1^ derived from an atomic model of the F-actin fibril and MPL of 15.4 kDa nm^−1^ determined using STEM^28^. The MPL of β_2_m amyloid fibrils have been calculated previously using STEM data. MpUL-multi determined an MPL of 56 ± 4 kDa.nm^−1^ (n = 31 fibrils, 14067 measurements; Fig. 4A), corresponding well to the previous estimate of 53 ± 3 kDa nm^−1^ for two-protofilament “type I” and “type II” β_2_m fibrils^13^.

Unlike fibrils of F-actin or β_2_m, no measurements of MPL for fibrils grown from full-length, wild type α-syn are currently available. We therefore also used MpUL-multi to make such measurements, yielding a MPL of 70 ± 2 kDa nm^−1^ (n = 23 fibrils, 20091 measurements; Fig. 4B) (Table 1).

An analysis of fluctuations in background intensity for the WT α-syn fibrils was performed as in^21^ to estimate the contribution to uncertainty in the MPL measurements. Background fluctuations were measured as:


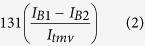


Where B_1_ and B_2_ are subsequent background measurements, one pixel apart. The standard deviation of the Gaussian distribution of background measurements was 10.5 compared to 16.1 for the background-subtracted fibrils. This is similar to values reported in Chen *et al.*^21^ but does suggest some contributions from other sources of error, which could include heterogeneity in the fibril structure along with other factors such as variability in the standards and effects during data collection such as beam induced mass loss.

We also determined the MPL of two variants of α-syn (A53T and A30P) that are known to occur naturally in individuals with early-onset Parkinson’s disease, and have been previously suggested to have fibril morphologies that vary from the WT fibrils (Table 1)^29^,^30^. α-syn A53T showed a MPL of 60 ± 4 kDa nm^−1^ (n = 29 fibrils, 11588 measurements), while α-syn A30P showed a MPL of 43 ± 3 kDa nm^−1^ (n = 25 fibrils, 14025 measurements). These estimates are consistent with very recent measurements of MPL for three different polymorphs of α-syn made using STEM^31^. Given the molecular mass of an α-syn monomer (14.4 kDa), this corresponds to 4.8 ± 0.2, 4.1 ± 0.3, and 2.9 ± 0.2 monomers per nm respectively, suggesting that these fibrils do indeed have different protofilament arrangements and/or architectures, information that can be used to guide model building in ongoing structural studies.

Vilar *et al.*^32^ proposed models for α-synuclein based of EM and solid-state NMR analysis of fibrils composed of the core α-syn_30-110_ fragment. Fibrils in this model are assembled from combinations of protofilaments having 2 protein molecules per β-sheet spacing. The estimated MPL values suggest the α-syn and α-syn A53T fibrils examined in this study would be single protofilaments of the Vilar *et al.* model. This may suggest the N- and/or C-terminal regions of α-syn play a role in the assembly of protofilaments into larger fibular aggregates.

The MpUL-multi program and GUI are designed to expedite the determination of fibril MPL and allow groups that do not have access to STEM to make rapid MPL estimations. TB-TEM dark field imaging is relatively easy to implement and does not require any special equipment beyond a standard TEM. The TB-TEM method is however, sensitive to ion contamination during grid preparation, especially from buffer salts. The TMV standard used should preferably be stored in pure water rather than a salt-containing buffer, water should be as pure as possible, and ions generated by interactions between the buffer and grids can be mitigated for by the use of inert titanium or gold grids. Freeze drying the sample to the grid may be preferable to air-drying as the sample may be sensitive to surface tension forces generated by the drying process. It is inherently less accurate than STEM measurements. However, it does allow estimates of mass per unit length to be made on in-house EM resources, and the MpUL provided GUI simplifies the process of selecting fibrils and standards. Finally, MpUL-multi performs image analysis and generates plain text measurement outputs for analysis as desired. Although not as accurate as MPL measurement by STEM, this allows for rapid ‘working estimates’ of fibril MPL within ~15%, allowing for identification of gross morphological differences that can be investigated by orthogonal biochemical and/or microscopy methods.

## Additional Information

**How to cite this article**: Iadanza, M. G. *et al.* MpUL-multi: Software for Calculation of Amyloid Fibril Mass per Unit Length from TB-TEM Images. *Sci. Rep.* **6**, 21078; doi: 10.1038/srep21078 (2016).

## Figures and Tables

**Table 1:** MPL values calculated for 5 fibular protein samples.

Fibril	Measured MPL	Literature MPL
β2M	56 ± 4	53 ± 3^1^
F-actin	17 ± 5	15.4 ± 1.9^2^
α-synuclein	70 ± 2	59.1, ~45^31^
α-synuclein A53T	60 ± 4	n/a
α-synuclein A30P	43 ± 3	n/a

1White *et al.*, 2009^13^.

^2^Steinmetz, *et al.*, 1998^28^.
